# The occurrence, characteristics, and adaptation of A-to-I RNA editing in bacteria: A review

**DOI:** 10.3389/fmicb.2023.1143929

**Published:** 2023-03-07

**Authors:** Weixue Liao, Wenhan Nie, Iftikhar Ahmad, Gongyou Chen, Bo Zhu

**Affiliations:** ^1^Shanghai Yangtze River Delta Eco-Environmental Change and Management Observation and Research Station, Shanghai Cooperative Innovation Center for Modern Seed Industry, School of Agriculture and Biology, Shanghai Jiao Tong University, Shanghai, China; ^2^Department of Environmental Sciences, COMSATS University Islamabad, Vehari Campus, Vehari, Pakistan

**Keywords:** RNA modification, adaptation, non-synonymous editing, ROS, Fe^3+^ uptake, TadA, adenosine deaminase

## Abstract

A-to-I RNA editing is a very important post-transcriptional modification or co-transcriptional modification that creates isoforms and increases the diversity of proteins. In this process, adenosine (A) in RNA molecules is hydrolyzed and deaminated into inosine (I). It is well known that ADAR (adenosine deaminase acting on RNA)-dependent A-to-I mRNA editing is widespread in animals. Next, the discovery of A-to-I mRNA editing was mediated by TadA (tRNA-specific adenosine deaminase) in *Escherichia coli* which is ADAR-independent event. Previously, the editing event S128P on the flagellar structural protein FliC enhanced the bacterial tolerance to oxidative stress in *Xoc*. In addition, the editing events T408A on the enterobactin iron receptor protein XfeA act as switches by controlling the uptake of Fe^3+^ in response to the concentration of iron in the environment. Even though bacteria have fewer editing events, the great majority of those that are currently preserved have adaptive benefits. Interestingly, it was found that a TadA-independent A-to-I RNA editing event T408A occurred on *xfeA*, indicating that there may be other new enzymes that perform a function like TadA. Here, we review recent advances in the characteristics, functions, and adaptations of editing in bacteria.

## 1. Introduction

RNA editing is a post-transcriptional modification or co-transcriptional modification mechanism that alters the sequence of RNA by inserting, deleting, or modifying nucleotides on the RNA molecule, and it can change the flow of genetic information (Srinivasan et al., 2021). The most prevalent type of RNA editing in organisms is A-to-I RNA editing, in which adenosine (A) converted to inosine (I) on RNA *via* adenosine deaminases. All adenosine deaminases are derived from a common bacterial ancestor and thus have similar catalytic subunits and conserved catalytic centers (Iyer et al., 2011).

A-to-I RNA editing events have been reported in tRNA from several domains. All deaminases have evolved from the bacterial ancestor enzyme TadA, and it is considered that ADAT1 contains the evolutionary clade of ADARs, while ADAT2 and ADAT3 belong to the evolutionary clade of the activation-induced deaminase (AID) or apolipoprotein B mRNA editing enzyme catalytic peptide-like (APOBEC) (Gerber and Keller, 1999; Jin et al., 2009; Iyer et al., 2011; Torres et al., 2014; Duan et al., 2022). In eukaryotes, the A-to-I RNA editing events are mediated by the ADAT1 homologous dimer at position 37 (A_37_) in the anticodon loop of tRNA^Ala^ (Grosjean et al., 1996; Gerber et al., 1998; Maas et al., 1999; Jühling et al., 2009). While it only exists in Archaea at position A_57_ of tRNA, whose catalytic enzymes are currently unknown (Yamaizumi et al., 1982; Grosjean et al., 1995, 1996). Unlikely editing at tRNA position A_57_, A-to-I editing events at tRNA wobble position A_34_ have been reported, which are mediated, respectively, by homologous dimer TadA (tRNA specific adenosine deaminase) in bacteria or heterologous dimer ADAT2/3 in eukaryotes. It is different that TadA only catalyzes at position A_34_ of tRNA^Arg^ in bacteria, while ADAT2/3 can catalyze eight of the tRNA substrates (seven substrates in *Saccharomyces cerevisiae*) (Gerber and Keller, 1999; Wolf et al., 2002; Jühling et al., 2009; Machnicka et al., 2013; Torres et al., 2014; Rafels-Ybern et al., 2018, 2019). Editing events at tRNA’s wobble position may expand the range of codon recognition because inosine (I) combines with uracil (U), cytidine (C), and adenosine (A) (Crick, 1966; Torres et al., 2014; Srinivasan et al., 2021). It is interesting that the edited tRNA^INN^ frequently correlates to the most preferred codon NNC in the organism, but there is no corresponding tRNA^GNN^, which indicates that the organism’s codon preference is mutually adapted to the position A_34_ of tRNA editing (Gerber and Keller, 1999; Rafels-Ybern et al., 2018, 2019; Lyu et al., 2020). Furthermore, particularly in eukaryotes with further expansion at position I_34_ of tRNA, the presence of editable position A_34_ of tRNA profoundly affects the composition of their tRNA genes, the genome-wide codon usage preference, and protein translation efficiency (Torres et al., 2021; Bertotti et al., 2022).

The phenomenon of A-to-I RNA editing was initially discovered in animals, which have been the most well researched (Bass and Weintraub, 1987, 1988; Rebagliati and Melton, 1987; Bass et al., 1989). Subsequent studies have shown that this ubiquitous process is mediated by the ADAR family in animals, whereas A-to-I mRNA editing events have not been found in other organisms lacking ADAR homologs (Nishikura, 2010; Yablonovitch et al., 2017). However, Liu and colleagues discovered ADAR-independent A-to-I mRNA editing events in filamentous fungi (*Fusarium graminearum*) for the first time in 2016, and they speculated that ADAT2/3 was responsible for the editing (Liu et al., 2016, 2017). Subsequently, Dan Bar-Yaacov and colleagues discovered the TadA-mediated A-to-I mRNA editing event for the first time in bacteria (*Escherichia coli*) (Bar-Yaacov et al., 2017). In our laboratory, we previously found TadA-dependent and TadA-independent A-to-I mRNA editing events in *Xanthomonas oryzae* pv. *oryzicola* (*Xoc*) (Nie et al., 2020, 2021). A-to-I mRNA non-synonymous editing events located in coding regions will directly alter genetic information due to inosine (I) being recognized as guanosine (G) during translation. This will enable the same gene to acquire the ability to express different isoforms of proteins and regulate the ratio of pre-edited and edited proteins to increase protein diversity at the post-transcriptional level, thereby refining gene expression regulation and improving potential adaptation (Gommans et al., 2009; Duan et al., 2017; Bar-Yaacov et al., 2018).

In fact, in terms of editing, substrate preferences, and adaptation, the A-to-I mRNA editing events in bacteria differ from those in animals and fungi. Next, we will review A-to-I mRNA editing events in bacteria and discuss their occurrence, regulation, and significance. We also look forward to further analysis and application of the editing mechanism in the future.

## 2. Characteristics and mechanism of A-to-I mRNA editing in bacteria

### 2.1. Secondary structure and recognition motifs differ between bacteria and fungi or animals

A-to-I RNA editing targets double-stranded RNA substrates in animals, which is also consistent with the fact that dsRNA-binding domains exist for the ADARs (Sommer et al., 1991; Nishikura, 2010, 2016; Grice and Degnan, 2015). And RNA editing seems to have only a weak preference for motifs near the editing site in animals, with depletion of G at the –1 position of A and enrichment of G or A at the +1 position (Eggington et al., 2011; Porath et al., 2017). While editing events both in fungi and bacteria have a strong motif preference and prefer A on the RNA hairpin loop. In fungi, mRNA editing strongly favors U at the –1 position of A, with A and G enriched at the +1 and +3 positions (Liu et al., 2016, 2017; Bian et al., 2019). Whereas in bacteria, it favors a greater UACG editing preference motif, which is present at all 15 editing sites in *E. coli* (Bar-Yaacov et al., 2017).

### 2.2. Enzymes that mediate A-to-I RNA editing in bacteria

In fungal and bacterial mRNA editing, the editing site’s preference for secondary structure and motif is very similar to tRNA editing. Liu and colleagues speculated that A-to-I mRNA editing events were mediated by ADAT2/3 rather than ADAT1 in fungi (Liu et al., 2016). While initial studies in bacteria have demonstrated that A-to-I mRNA editing in *E. coli* is mediated by TadA, which is responsible for tRNA^Arg^ editing, overexpression or silencing of *tadA* results in a corresponding change in the number of editing sites and the level of editing (Bar-Yaacov et al., 2017). Consistent with this, a previous study from our lab demonstrated that the site of a serine to proline mutation (S128) editing event on the *fliC* in *Xoc* was also catalyzed by TadA，with corresponding rise and disappearance after *tadA* overexpression and knockdown (Nie et al., 2020). There is a noteworthy point that the ICG-tRNA^Arg^ is edited by TadA, which is the only tRNA that can recognize the most preferred codon CGC of Arg in either *E. coli* or *Xoc* (Lyu et al., 2020). The *tadA* has been shown to be an essential gene in *E. coli* (Wolf et al., 2002). Interestingly, based on the OGEE (Online Gene Essentiality) database (Gurumayum et al., 2021), it found the *tadA* was a nonessential gene in some typical strains such as *Bacillus subtilis* subsp. *subtilis* str. 168 (BSU00180) and *Pseudomonas aeruginosa* PAO1 (PA4302) (Nie et al., 2020). Besides, *tadA* can be completely knocked out in *Xoc*, which suggests the existence of an isoenzyme of TadA in *Xoc*. It is consistent that A-to-I editing events at position A_34_ of tRNA^Arg^ are still observed in RNA-seq data of the knockout *tadA* mutant (Nie et al., 2021). In addition, we demonstrated that the A-to-I mRNA editing event on *xfeA* leading to a threonine to alanine mutation (T408A) is independent of *tadA* in *Xoc*, but the editing site has the same secondary structure and most preferred motif UACG as the TadA-independent A-to-I mRNA editing events (Nie et al., 2021). The result indicates that the TadA isozyme may be involved in A-to-I mRNA editing in *Xoc*.

### 2.3. The relationships between A-to-I editing of mRNA and tRNA

It is a general fact that ADATs only catalyze tRNA substrates but no other RNAs in the most organisms. This general conservation of substrate restriction means that the catalytic effect of ADATs on other RNAs may be inhibited by selective pressure. It is assumed that ADATs have acquired the ability to randomly edit mRNA due to an individual mutation during evolution. For example, in order to achieve directed evolution, some amino acid mutations in TadA can be subjected to directed evolution so that TadA can edit more kinds of genes (Gaudelli et al., 2017). The editing results may disturb the transmission of genetic information on a large scale, which will reduce the fitness of the organism and even cause serious developmental defects directly. As a result, most organisms’ ADATs are only specific in identifying and editing their tRNA substrates when under selection pressure. An interesting question is how, if A-to-I mRNA editing is mediated by TadA in bacteria, they would recognize mRNA for editing if they were previously specific in identifying tRNA. Studies have shown that the recognition of tRNA by ADAT2/3 depends on its tertiary structure, whereas TadA recognizes its unique tRNA substrate through motifs and secondary structure (Losey et al., 2006; Frigole et al., 2019; Liu et al., 2020; Ramos-Morales et al., 2021; Dolce et al., 2022). However, bacterial A-to-I mRNA editing events have higher motif requirements, as opposed to seven or eight substrates for enzymes that mediate editing in eukaryotes, the vast majority of editing sites have the same UACG editing motif and secondary structure in the tRNA^Arg^ as the only substrate of TadA in bacteria (Bar-Yaacov et al., 2017), which indicates that A-to-I mRNA editing events may be accidental off-target events of TadA on tRNA due to similar substrates in bacteria. Individual editing events with adaptive significance have been preserved throughout evolution, which can explain why bacteria have so few mRNA editing events. In general, at present, the enzymes edited by bacterial A-to-I mRNA and tRNA are both mediated by TadA, and the substrates recognized by the enzymes are similar in structure and motif.

## 3. The function and meaning of A-to-I RNA editing in bacteria

A-to-I RNA editing plays many roles in the regulation of biological functions in bacteria. Dan Bar-Yaacov and colleagues previously discovered TadA-dependent A-to-I mRNA editing in *E. coli* and demonstrated the effect of A-to-I RNA editing on the *hokB* (host-killing toxins B) (Bar-Yaacov et al., 2017). Verstraeten and colleagues demonstrated that GTPase Obg induces multidrug resistance at the single-cell level by activating the toxin *hokB* in a (p)ppGpp-dependent manner (Verstraeten et al., 2015). Therefore, it is speculated that editing events on *hokB* are associated with bacterial tolerance to antibiotics in *E. coli*. In addition to the A-to-I RNA editing event occurring on tRNA-Arg in *E. coli*, the study found that there were 15 novel A-to-I editing events on mRNA, and 12 of them occurred in the coding region, and all are missense edits that recode tyrosine codons (UAC) to cysteine codons (UGC) (Bar-Yaacov et al., 2017). It is worth noting that 4 of the 12 missense editing events are enriched in the *hok* family genes; and the average editing level of the editing sites located in the *hokB* gene is 76%, which is also the editing level of the sites with the highest levels of mRNA editing (Bar-Yaacov et al., 2017). *hokB* is a gene encoding a toxin that can limit the growth of cells by expressing it. Since many antibiotics have toxic effects on dividing cells, regulating the expression of *hokB* may have a potential impact on bacterial antibiotic resistance to varying degrees (Pedersen and Gerdes, 1999; Verstraeten et al., 2015; Harms et al., 2016). It is known that the transcriptional level of *hokB* is controlled by a high level of alarmone (p) ppGpp (Gerdes and Maisonneuve, 2015). It is interesting to find that missense editing events can alter the toxicity of *hokB*. Furthermore, it discovered the growth of the body in bacterial culture and found that the edited *hokB* is more virulent by comparing the pre-edited and edited versions of *hokB* mutations. In fact, the editing level of *hokB* in *E. coli* is variable and adjusts the proportion of edited versions during the growth process, which means that A-to-I RNA editing can further refine the degree of action of *hokB* in cells by regulating the editing level to change the toxicity of *hokB* at the post-transcriptional level. From the above, it can be inferred that all of these can improve the adaptation of populations to various antibiotic stresses by increasing the richness of antibiotic-resistant subgroups in the bacterial population (Bar-Yaacov et al., 2017, 2018).

Reactive oxygen species (ROS) exist in the natural environment and in plants. ROS production is also a plant defense response to pathogens that infect them (Torres et al., 2006; Benedetti et al., 2015). Long-term exposure of pathogens to the oxidative environment of ROS is not conducive to their survival (Imlay, 2013). Therefore, the pathogens will adopt some strategies to overcome oxidative stress, such as protozoa, which can overcome oxidative stress by producing some ROS-tolerant enzymes (Fones and Preston, 2012). The structure, formation, and movement of biofilms are closely related to the formation of flagella. Changes in the structure of the flagella can affect the bacteria’s ability to form biofilms and overcome ROS (Asahi et al., 2015; Tian et al., 2015; Buscaill et al., 2019). Through transcriptome sequencing and bioinformatics analysis, our lab previously found that there is a non-synonymous A-to-I mRNA editing event (S128P) mediated by TadA on the filament structural protein FliC in *Xoc* under H_2_O_2_ treatment. Experimental data indicated that overexpression or knockout of *tadA* resulted in a corresponding increase and disappearance of editing levels at this site under H_2_O_2_ treatment. Furthermore, iRIP-seq data revealed that *tadA* enrichment exists for *fliC* mRNA (Nie et al., 2020). But one of difference from *E. coli* is that the editing site on *filC* has a GACG motif instead of the UACG motif mentioned in previous studies. In fact, the editing sites of non-UACG motifs such as AACG, UAUG, CACG, GACG, and UAAG also appear in *E. coli* overexpressing *tadA* mutants (Bar-Yaacov et al., 2017). We speculate that the promiscuous A-to-I RNA editing motif in the *Xoc* strain is related to ROS stress.

The editing event on the flagellar filament protein FliC only occurs at position 128 (Nie et al., 2020). There was no significant difference in the growth of the two versions of pre-edited and edited in the absence of H_2_O_2_. However, the growth of the pre-edited version was slower than that of the edited version in the presence of H_2_O_2_, and the edited version was more resistant to H_2_O_2_. In general, editing enhances the tolerance of strains to oxidative stress. By measuring the flagellar motility and adhesion in the two pre-edited and edited versions of mutants of the S128P editing event, it was found that the edited version of the strain had a longer flagella length and was calculated to have greater traction in motion. At the same time, the edited strains have higher adhesion ability and biofilm formation ability, which play a significant role in resisting oxidative stress (Nie et al., 2020). The underlying reason for this phenotype may be that S128P is located in the D1 domain of the predicted FilC structure, which is highly conserved in bacteria (Song and Yoon, 2014). The S128P editing event results in a weakened interaction between the conserved domain D1 of FliC protein and the hypervariable domains D2 and D3, which in turn leads to changes in flagellar structure and thus may alter flagellar motility and adhesion (Nie et al., 2020).

Since editing leads directly to changes in flagellar filament structure, it does not yet fully explain the increased virulence of the edited version of the strain. What is the cause of the edited version’s virulence enhancement of the strain? We discovered that the amino acid change at *filC* S128 produced by editing may alter the expression of other genes *via* transcriptome sequencing and analysis of the edited and pre-edited strains. By comparing the transcriptomes of the two versions of S128P (edited and pre-edited strains), it was found that the *XOC_3386*-*XOC_3,390* gene cluster was up-regulated in the edited strain. Based on the functional annotation of the KEGG database, it was found that the up-regulated expression of this gene cluster was helpful for bacterial Fe^3+^ uptake and siderophore synthesis in the environment (Nie et al., 2020). Fe^2+^ and ROS can cause the Fenton reaction (Fenton, 1894). Studies have shown that OH▪ is the main ROS that causes oxidative damage (Vattanaviboon and Mongkolsuk, 1998), and the uptake of exogenous Fe^3+^ can reduce the OH▪ in *Xoc*, which indicates that the uptake of exogenous Fe^3+^ infusion helps *Xoc* resist H_2_O_2_ oxidative stress (Nie et al., 2020). Therefore, *fliC* S128P editing indirectly enhances the expression of the *XOC_3386*-*XOC_3,390* gene cluster and increases the uptake of Fe^3+^ in *Xoc*, which in turn reduces the ROS level in the bacteria, thus further improving the resistance of the bacteria to oxidative stress.

In conclusion, we found that the S128P editing event of *filC* enhanced the ROS tolerance by altering the flagellar structure directly to enhance the biofilm formation ability and indirectly by increasing the Fe^3+^ uptake level and reducing the OH▪ content. The enhancement of ROS is undoubtedly harmful to the infection of *Xoc*. After inoculating the edited mutant in rice leaves, it was found that the length of the lesions caused by the edited mutant is longer than that of the wild type and pre-edited. It shows that the virulence of the edited pathogenic bacteria is enhanced, and it can resist oxidative stress to a greater extent and reduce the accumulation of ROS. As a result, the edited mutant has improved infectivity and colonization of plant tissues, which is the result of mutual adaptation between the bacteria and their environment (including the host plant) (Nie et al., 2020).

Transition metals play an important role in the normal life activities of organisms. Appropriate metal ion concentrations help to improve the catalytic reaction activity in organisms (Hood and Skaar, 2012). However, it will also cause certain toxic effects on organisms when the concentration of transition metals is too high. As a result, effective metal ion concentration regulation *in vivo* is critical for the organism’s survival and reproduction, as it must not only satisfy the needs of its own growth activities but also prevent the harm caused by high metal concentrations.

Iron is a very important element, and sufficient iron is critical for bacterial pathogens to infect their hosts (Skaar, 2010; Franza and Expert, 2013). In the case of iron deficiency, being able to obtain sufficient iron from the outside world is of great significance for pathogenic bacteria to maintain the stability of invasion. Siderophore is very important for bacteria to uptake Fe^3+^, which forms a complex by chelating Fe^3+^ *in vitro* and then importing Fe^3+^ into the body through specific receptors. Enterobactin is one of the most important siderophores in gram-negative bacteria, and the formation of enterobactin (ferric enterobactin, Fe-Ent) occurs between enterobactin and Fe^3+^ in *Xoc*. Then, TonB-dependent receptors recognize Fe-Ent at the outer membrane and transport it to the periplasmic space, where it transports Fe^3+^ into the cell (Ferguson and Deisenhofer, 2002). Interestingly, previous studies found that there is non-synonymous A-to-I RNA editing at the T408 site (T408A) of the enterobactin iron receptor protein XfeA through transcriptome sequencing and bioinformatics in *Xoc*. The XfeA protein is homologous to the Fe-Ent outer membrane receptor protein FepA in *E. coli*, which plays an important role in the process of Fe-Ent sequestration of Fe^3+^ (Buchanan et al., 1999; Nie et al., 2021).

Versions of pre-edited, edited, and wild type were treated with iron chelators and iron supplements (FeCl_3_) to generate environments with various iron concentrations. It was discovered that editing levels increase as iron concentration decreases. It suggests that iron deficiency can increase A-to-I RNA editing at the *xfeA* T408A location while having no effect on *xfeA* transcription, implying that A-to-I RNA editing may be involved in the regulation of iron uptake (Nie et al., 2021). There was no significant difference in the growth of the pre-edited and edited strains in the absence of iron chelators and iron supplements (FeCl_3_). The pre-edited strains had longer growth lags and slower growth rates relative to the edited strains in the presence of iron chelators at concentrations of 100 μM and above. By measuring the intracellular iron concentration of the strains, it was found that the edited version of the strain had a higher intracellular iron concentration than the pre-edited version of the strain in both iron-deficient and iron-supplemented environments. It demonstrates that T408A editing in *xfeA* can improve the strain’s ability to uptake iron as well as its tolerance to iron-deficient environments (Nie et al., 2021).

So, how does T408A editing in *xfeA* improve the iron uptake of the strain? The obvious reason is that editing can change the type of protein and thus directly affect its function. According to 3D homology modeling prediction, it is found that the modification of the protein structure following the editing of the T408A site of *xfeA* may be beneficial to the binding of the protein and the ligand, so it is speculated that the T408A editing event of *xfeA* will affect the Fe-Ent binding outer membrane receptor protein efficiency, thereby improving the uptake capacity of Fe^3+^ in the strain (Nie et al., 2021).

Further, to prove that the edited strain has a higher iron-absorbing ability, it was found that *Xoc* had a higher chemotaxis response to iron when edited according to an analysis of the transcriptomes between the pre-edited and edited versions. It revealed that differentially up-regulated genes in post-editing mutants were highly correlated with chemotactic genes by comparing them with pre-editing mutants, including methyl-accepting chemotaxis proteins (MCPs) such as *XOC_2282* and *XOC_2291*, as determined by sequence analysis and functional annotation based on KEGG (Nie et al., 2021). Molecular interaction analysis confirmed that Fe-Ent directly binds to *XOC_2282* and *XOC_2291*, which may further enhance the expression of other related chemotactic genes (Newton et al., 1999; Ma et al., 2007; Wuichet and Zhulin, 2010). The chemotaxis of the pre-edited version of the strains to attractants (glucose, serine, Fe-Ent, and phosphate buffer) relative to glucose was measured using capillary chemotaxis experiments (Verma et al., 2018), and it was found that the T408A edited strain was stronger than the pre-edited version. Fe-Ent induces a more pronounced chemotactic response, suggesting that the *xfeA* T408A editing event enhances the bacterial chemotactic response to Fe-Ent, which in turn facilitates bacterial uptake of iron (Nie et al., 2021).

Iron uptake and chemotaxis are critical for strain pathogenicity in bacteria (Qian et al., 2009; Muok et al., 2019), and most bacteria deal with iron abundance and shortage *via* the iron uptake factor (Fur) (Hantke, 2001; Troxell and Hassan, 2013). In recent years, Fur-independent iron homeostasis regulation mechanism was discovered in bacteria (Wang et al., 2016). In previous studies in our lab, A-to-I RNA editing on *xfeA* T408A may be a Fur-independent iron homeostasis regulation mechanism. There is no doubt that this iron homeostasis regulation mechanism is significant for the pathogenicity of pathogens. It is consistent that rice leaf inoculation experiments showed that A-to-I RNA editing at T408A could enhance colonization and virulence in rice leaves (Nie et al., 2021). In general, A-to-I RNA editing at the T408A gene facilitates Fe-Ent entry into the periplasmic space and elicits MCP responses, which can enhance the ability of bacteria to uptake iron under iron-deficiency stress (Nie et al., 2021).

Bacterial mRNA editing was initially thought to be caused by the off-target event of TadA at tRNA. Therefore, when bacteria faced different adversities, the genes related to editing caused by random off-target events were also varied, thus giving bacteria various anti-adversity functions. As previously reported, a TadA-dependent A-to-I RNA editing event in *fliC* S128P that encodes the flagellar structural protein in *Xoc* changes the shape of the flagella and uptake of Fe^3+^ under oxidative stress. When bacteria infect host plants, indirect regulation of associated gene clusters allows them to resist ROS stress, and the current study explains the A-to-I RNA editing events that adapt to phytopathogenic bacteria under iron deficiency stress (Nie et al., 2021). Interestingly, the A-to-I RNA editing event is TadA-independent at the *xfeA* T408A because the A-to-I RNA editing event at the *xfeA* T408A still occurs even though *tadA* is knocked out. The recognition motif UACG of the *xfeA* T408A editing site was discovered to be similar to TadA-independent A-to-I mRNA editing events, which indicates that there may be a TadA-like adenosine deaminase that plays the role of mRNA editing (Nie et al., 2021).

## 4. Adaptive advantage of A-to-I RNA editing in bacteria

Non-synonymous mRNA editing causes a gene to produce multiple protein isoforms. What implications might such a finding have for an organism? It is simple to grasp that if the new protein created by editing provides greater adaptation for the organism than the pre-edited protein, then mutation will increase the adaptation of the editing site in the selection process. If the editing event is harmful, the editing level will gradually decrease and eventually disappear. This also reflects the distinction between RNA editing and DNA mutation. Editing provides the raw materials for evolution as well as the opportunity for organisms at the RNA level to experiment. According to the findings, non-synonymous editing in coleoid cephalopods is more likely to be restorative editing, in which the editing is used to restore harmful G to A mutations in the genome at the RNA level (Jiang and Zhang, 2019). Although, it has a positive restoring effect on current harmful mutations and is adaptable to its unmutated ancestor, this editing event cannot completely restore the mutation to its premutation state due to the editing level limitation. As previously stated, the final effect may be an A to G reverse mutation, but the entire process does not improve the organism’s adaptation (Jiang and Zhang, 2019). Additionally, a recent study found that nonsynonymous C-to-U RNA editing is adaptive due to its restorative effects in plants (Duan et al., 2023). On the other hand, some researchers believe that editing is adaptive because it has been discovered that even restored editing events are less prone to mutation than other A sites, which implies that it is evolutionarily deliberate to keep these events rather than reverting to their original state (Liu et al., 2017; Shoshan et al., 2021). These edits have the potential for adaptation. One possible adaptation is that distinct protein isoforms have functional variations and can adapt to diverse environments, whereas the editing level can directly affect the expression ratio of protein isoforms. Thus, affecting the editing level of the corresponding editing event can increase the proportion of adaptive protein isoforms traversing the associated environment, which improves the organism’s adaptation to this environment. This also means that editing events can increase protein diversity as well as the editing level involved in gene expression regulation.

Research shows that both the editing events on *hokB* in *E. coli* and the editing events on *fliC* and *xfeA* reflect the adaptation in bacteria. A high level of alarmone (p) ppGpp regulates *hokB* transcription. And then, the editing level can coordinate the transcriptional level to further regulate *hokB* toxicity, resulting in different growth inhibition states of the subpopulations of cells expressing *hokB*, which may allow the populations to acquire a potential for tolerance to various antibiotics (Bar-Yaacov et al., 2017). The S128P editing event in *fliC* also provides functional variety to the protein by modifying flagellar shape and biofilm formation, as well as increasing gene clusters that affect Fe^3+^ uptake under oxidative stress, which enhances bacterial tolerance to reactive oxygen species (Nie et al., 2020). The T408A editing event of *xfeA* rather than the transcriptional regulation of *xfeA* works as a programmable switch, regulating the intake of Fe^3+^ by bacteria in response to the concentration of Fe^3+^ in the environment (Nie et al., 2021). All of these editing processes in bacteria show the adaptive importance of editing in the face of adversity, and editing has even become a key mechanism for modulating gene function. In addition to the reason that strict substrate recognition by editing enzymes may lead to fewer editing sites in bacteria, it is also possible that this is due to bacteria’s short life cycles, fast reproduction, and sharp intraspecific competition. Because non-adaptive editing sites can be quickly replaced by genetic alterations due to internal competition, the vast majority of currently retained editing events could be adaptive (Rieder et al., 2015; Bar-Yaacov et al., 2017; Jiang and Zhang, 2019; Nie et al., 2020, 2021; Shoshan et al., 2021).

Individual editing events in single-cell bacteria are limited compared to multicellular creatures, and so they cannot endure every adversity, but differences between individuals in a community can expand the diversity of editing events in a population. Distinct cell subsets may have different editing events and levels, allowing for quick adaptation to a variety of adverse conditions and improving species fitness at the population level. In addition, we found that the *Pseudomonas putida* model strain KT2440 has an A-to-I editing event S491P in the *fliC* gene after exposure to H_2_O_2_, and the S491P edited mutant strain showed stronger tolerance to ROS than the wild-type strain KT2440. Considering the conservation of the editing enzymes, A-to-I mRNA editing may be widespread in bacteria, and mutations in DNA can replace non-adaptive editing sites. Other editing events may be hidden in the general growth environment but can only be detected in specialized conditions. Given that bacteria are one of the most common diseases in animals and plants, a deeper understanding of A-to-I RNA editing is essential for harmful bacteria to resist host immunity and antibiotic stress, which is significant to further understand the pathogenic mechanism and discover drug targets.

## 5. Conclusion

We concentrated on our earlier studies while reviewing the incidence, function, and adaptation of bacteria. Both the S128P and T408A A-to-I RNA editing events in *Xoc* have been shown to improve the bacteria’s ability to resist environmental stress. The presence of the TadA-independent editing event *xfeA* T408A suggests the presence of other adenosine deaminases. In the future, we will focus on the discovery of new RNA editing enzymes and find these targets in the signal transduction process with more direct molecular biological evidence. We also explore the function of the editing sites in more bacterial species, further explaining how A-to-I RNA editing is regulated in bacteria.

## Author contributions

BZ and WL contributed to conception and design of the study. WL and WN wrote the first draft of the manuscript. BZ, IA, and GC revised the manuscript. BZ supervised the project. All authors contributed to manuscript revision, read, and approved the submitted version.

## Funding

This work was supported by National Natural Science Foundation of China (32272479, 31200003), Open Project Program of State Key Laboratory of Rice Biology (20190109), Open Project Program of State Key Laboratory for Biology of Plant Diseases and Insect Pests (SKLOF202201), Shanghai Committee of Science and Technology (19390743300 and 21ZR1435500), Chongqing Natural Science Foundation (CSTB2022NSCQ-MSX0524), and Zhejiang Province ecological Environment Research and Promotion Project (2020HT0009).

## Conflict of interest

The authors declare that the research was conducted in the absence of any commercial or financial relationships that could be construed as a potential conflict of interest.

## Publisher’s note

All claims expressed in this article are solely those of the authors and do not necessarily represent those of their affiliated organizations, or those of the publisher, the editors and the reviewers. Any product that may be evaluated in this article, or claim that may be made by its manufacturer, is not guaranteed or endorsed by the publisher.
